# Increased thalamic gray matter volume induced by repetitive transcranial magnetic stimulation treatment in patients with major depressive disorder

**DOI:** 10.3389/fpsyt.2023.1163067

**Published:** 2023-05-12

**Authors:** Zhongheng Wang, Dongning Zhang, Muzhen Guan, Xiaojiao Ren, Dan Li, Kaiming Yin, Ping Zhou, Baojuan Li, Huaning Wang

**Affiliations:** ^1^Department of Psychiatry, Xijing Hospital, Air Force Medical University, Xi'an, China; ^2^Department of Mental Health, Xi'an Medical College, Xi'an, China; ^3^Department of Psychiatry, Yulin Fifth Hospital, Yulin, China; ^4^Department of Psychiatry, Shi Jiazhuang Psychological Hospital, Shijiazhuang, China; ^5^School of Biomedical Engineering, Air Force Medical University, Xi'an, China

**Keywords:** major depressive disorder, repetitive transcranial magnetic stimulation, voxel based morphometry, gray matter volume, thalamus

## Abstract

**Purpose:**

Repetitive transcranial magnetic stimulation (rTMS) is an effective therapy in improving depressive symptoms in MDD patients, but the intrinsic mechanism is still unclear. In this study, we investigated the influence of rTMS on brain gray matter volume for alleviating depressive symptoms in MDD patients using structural magnetic resonance imaging (sMRI) data.

**Methods:**

Patients with first episode, unmedicated patients with MDD (*n* = 26), and healthy controls (*n* = 31) were selected for this study. Depressive symptoms were assessed before and after treatment by using the HAMD-17 score. High-frequency rTMS treatment was conducted in patients with MDD over 15 days. The rTMS treatment target is located at the F3 point of the left dorsolateral prefrontal cortex. Structural magnetic resonance imaging (sMRI) data were collected before and after treatment to compare the changes in brain gray matter volume.

**Results:**

Before treatment, patients with MDD had significantly reduced gray matter volumes in the right fusiform gyrus, left and right inferior frontal gyrus (triangular part), left inferior frontal gyrus (orbital part), left parahippocampal gyrus, left thalamus, right precuneus, right calcarine fissure, and right median cingulate gyrus compared with healthy controls (*P* < 0.05). After rTMS treatment, significant growth in gray matter volume of the bilateral thalamus was observed in depressed patients (*P* < 0.05).

**Conclusion:**

Bilateral thalamic gray matter volumes were enlarged in the thalamus of MDD patients after rTMS treatment and may be the underlying neural mechanism for the treatment of rTMS on depression.

## Introduction

Major depressive disorder (MDD) is a common psychiatric disorder characterized by persistent depressed mood, decreased interest, and cognitive impairment (1), with high morbidity, relapse, and mortality rate (2). More and more scholars have initiated to study the structural and functional brain alterations in MDD by using various emerging magnetic resonance techniques (3), aiming to provide essential objective imaging indicators for the early diagnosis of depressive disorder and to help clarify the pathogenesis of depressive disorder as well (4).

In 2000, Ashburner and Friston originally proposed the voxel-based-morphometry (VBM) method for the evaluation of the structural alterations in the gray matter of the brain (5). It is an objective, comprehensive, and automated means of analyzing brain magnetic resonance images based on voxel levels to quantify changes in the density and gray and white matter volume in relevant brain regions and to provide an accurate morphological analysis of the brain tissue structure (6). It was found that compared to healthy subjects, patients with first episode and unmedicated depressive disorder showed significant reductions in the gray matter in the dorsolateral prefrontal cortical area, dorsomedial prefrontal cortical area (DMPFC), and ventral lateral prefrontal cortical area (VLPFC). Compared to patients in remission from depressive disorder, their dorsolateral prefrontal cortical area, ventral lateral prefrontal cortical area, precuneus, inferior parietal lobule, and anterior cingulate cortex (ACC) all demonstrated a decline in gray matter structures (7).

The treatment of depressive disorder is primarily pharmacological, with antidepressant medication often starting to work after 2 weeks and a minimum of 4 weeks to determine whether the medication is effective, such as venlafaxine (8, 9). A total of 20–30% of the patients with major depressive disorder (MDD) do not respond to their first pharmacological and psychological treatment (10, 11). For patients with MDD who do not respond well to clinical first-line medication or who cannot tolerate the side effects of antidepressants (12), repetitive transcranial magnetic stimulation (rTMS) has gained attention in the antidepressant field as a noninvasive and well-tolerated physical therapy (13). Boes et al. proved that rTMS could increase the cortical thickness of the rostral anterior cingulate cortex (rACC). The cortical thickness values of the rACC vs. dorsal anterior cingulate cortex (dACC) predicted the effect of rTMS in the treatment of MDD as well (14). Lan et al. discovered that rTMS increased the gray matter density in the mPFC (15). Hayasaka et al. found that rTMS enlarged the gray matter volume in the hippocampus (16). In contrast, the results from the study conducted by Furtado et al. demonstrated that rTMS causes further shrinkage of gray matter volume in the left hippocampus of MDD patients (17).

At present, studies exploring the effects of rTMS on the gray matter structure of the brain in MDD are scarce, and the findings are fragmented and inconsistent. Nonetheless, the primary contribution of this study is the correlation between these anomalies and the effects of rTMS clinical treatments In this study, sMRI data were collected to compare the differences in gray matter volume between MDD patients before and after rTMS treatment and to explore the potential neural mechanisms of rTMS to improve depressive symptoms.

## Methods

### Participants

We performed a single-blind, randomized, control study based on the medical records of 26 patients with first episode and unmedicated depressive disorder consecutively from August 2020 to December 2021 from the outpatient clinic of the Department of Psychiatry, the First Affiliated Hospital, Air Force Medical University. Meanwhile, 31 healthy adults were recruited as controls.

### Inclusion and exclusion criteria

The inclusion criteria of the research subjects were as follows: (1) meeting the diagnostic criteria for MDD in DSM-5; (2) 18–45 years old; (3) HAMD-17 scores ≥18; (4) right-handedness, normal physical examination, EEG examination, and other indicators; and (5) those who signed informed consent. The exclusion criteria were as follows: (1) those suffering from severe physical diseases; (2) those with a previous history of traumatic brain injury or brain surgery; (3) those with a history of alcohol or substance abuse; (4) those with a history of other psychiatric or neurological disorders; (5) those with a high risk of suicide; (6) pregnant or lactating women; and (7) those with contraindications to MRI.

The inclusion criteria for healthy controls were (1) ages from 18 to 45 years and (2) right-handedness. The exclusion criteria were as follows: (1) severe physical illness; (2) previous history of traumatic brain injury or brain surgery; (3) history of alcohol or substance abuse; (4) history of psychiatric illness in patients; (5) pregnant or lactating women; and (6) contraindication to MRI.

### Ethics approval

The study was approved by the Ethics Committee of the First Affiliated Hospital of Air Force Medical University (Grant No. KY20202055-F-1). All subjects both read and understood the trial procedures and precautions and then signed the informed consent form to authorize participation.

### Randomized controlled trial

A randomized controlled method was conducted. The computer Excel software was used to allocate random numbers to patients using a uniform distribution program, and finally, 26 patients were enrolled in the project.

### Treatments

#### Repeated transcranial magnetic stimulation treatment

The Transcranial Magnetic Stimulation Therapy Instrument (CCY-1) (Yiruide Group, Wuhan, China) (figure-of-eight coil) was used to treat depressive patients with rTMS for 15 consecutive days, starting on the day of enrollment. The stimulation frequency was 10 Hz, and the left dorsolateral prefrontal cortex (positioned with the international 10–20 system, corresponding to the electrode point F3) was used as the stimulation target. The intensity was 110% of the motor threshold, and each sequence consisted of 23 treatment sequences of 50 pulses with an interval of 35 s, 1,150 pulses in total (10 pulses per second, lasting for 5 s). The treatment was given once a day for 15 days.

#### Drug treatment

Patients with MDD were given venlafaxine hydrochloride sustained-release capsules (Pfizer Prozac H20160382, specification: 75 mg) at a starting dose of 75 mg/day orally once daily in the morning, starting from the day of rTMS treatment. During rTMS treatment, the treatment dose was gradually titrated according to the patients' drug response and drug resistance, and the effective dose of 150 mg/day was reached within 15 days. All subjects maintained a regular diet and a stable drug dose throughout the study period.

### Data collection

#### Clinical assessment

The 17-item Hamilton depression scale (HAMD-17) was used to assess depressive symptoms in the patient and control groups (18). The HAMD-17 assessment was performed at the baseline and on the 15th day of rTMS treatment in the patient group and on the day of enrollment in the control group.

#### Magnetic resonance data acquisition

Scans were performed on the day of enrollment and on the 15th day after treatment for depressive patients and on the day of enrollment for healthy controls. A General Motors 3.0 T magnetic resonance imaging system (GE MR 750, USA) with an 8-channel head coil was used for signal reception. A 3D Bravo T1-weighted scan was performed with the following scan parameters: TR = 8.1 msx, TE = 3.2 ms, matrix = 256 × 256, layer thickness = 1 mm, and FA = 12. A total of 176 layers were scanned to cover the whole brain, using the posterior joints as the scan baseline.

#### VBM data processing analysis

The brain structure images were processed based on Matlab2018b and the VBM toolkit (http://dbm.neuro.uni-jena.de/vbm8) in SPM8 (https://www.fil.ion.ucl.ac.uk/spm/software/spm8/). The images were manually corrected based on the anterior and posterior commissure. The structural images of the subjects were segmented into gray matter, white matter, and cerebrospinal fluid by the default segmentation algorithm. The gray matter images were further aligned to MNI standard space using the DARTEL algorithm. Spatial smoothing was applied with an 8-mm full-width half-height Gaussian kernel, and the smoothed gray matter images were used for comparative analysis.

### Statistical analysis

After pre-treatment, structural images were statistically analyzed using a general linear model with age, gender, education, and cranial volume as covariates and gray matter voxels as the basis. Furthermore, a two-sample *t*-test was used for comparison between the two groups, and a paired-sample *t*-test was used for comparison between patient groups before and after treatment, and the differences were considered statistically different after FDR correction with voxels > 50 and *P* < 0.05. The gray matter volume of different brain regions was extracted, and their correlations with HAMD-17 scores were analyzed. A *p*-value of <0.05 was considered to be statistically significant.

## Results

### Analysis of general demographic data

The study was divided into pre-treated MDD patients, post-treated MDD patients, and healthy controls. Among 26 cases enrolled in the MDD treatment group, nine were men and 17 were women. The mean age was 26.81 ± 8.24 years (ranging from 18 to 45 years), and the average disease duration was 5.11 ± 3.62 months. In the healthy control group, 11 patients were men and 20 were women, with 31 in total. The mean age was 28.72 ± 9.03 years (ranging from 18 to 45 years). There were no significant differences in age and gender composition between the two groups (*P* > 0.05; Table 1).

**Table 1:** Demographic information of two groups of subjects.

	**MDD group (*n* = 26)**	**Control group (*n* = 31)**	***P***
Mean age	26.81 ± 8.24	28.72 ± 9.03	0.55
Gender (F/M)	17/9	20/11	0.83
Duration of education	13.29 ± 3.27	15.72 ± 2.36	0.39

MDD, major depressive disorder; F, female; M, male.

The difference was considered statistically significant if the P-value was < 0.05.

### Assessment of the efficacy of depressive symptoms

The total HAMD-17 scores of depressive patients were significantly higher than those of healthy controls (*P* < 0.001), and the total HAMD-17 scores of MDD patients after treatment were significantly lower than the total scores before treatment (*P* < 0.001; Table 2).

**Table 2:** Comparison of total HAMD-17 scores between MDD patients and healthy controls.

	**MDD group (*n* = 26)**	**Control group (*n* = 31)**	***t***	***P***
Pre-rTMS	20.88 ± 7.05	3.74 ± 2.61	14.103	0.000
Post-rTMS	13.46 ± 4.28	-	6.864	0.000

### Results of gray matter volume data

**Figure 1:**
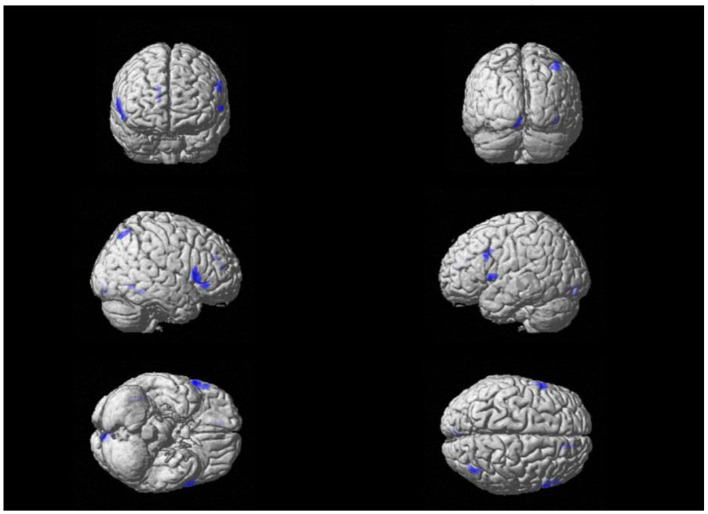
Volume-reduced gray matter in different brain regions in MDD patients and healthy controls.

By analyzing the MRI images of the brains of MDD patients and healthy controls, gray matter volumes of the right fusiform gyrus, left and right inferior frontal gyrus (triangular part), left inferior frontal gyrus (orbital part), left parahippocampal gyrus, left thalamus, right precuneus, right calcarine fissure, and right median cingulate gyrus were significantly reduced in the MDD patient group before treatment compared with the healthy control group (*P* < 0.05). The results are detailed in Table 3 and Figure 1.

**Table 3:** Comparison of healthy control and pre-treatment gray matter volumes.

**Brain regions**	**BA**	**Side**	**MNI coordinates**			**Cluster size**	***t***	***P***

			**X**	**Y**	**Z**			
Fusiform gyrus	30	Right	17	−32	−21	197	11.36	0.000
Inferior frontal gyrus (triangular part)	48	Left	−57	12	3	138	8.97	0.000
Inferior frontal gyrus (orbital part)	38	Left	−50	26	−9	169	8.88	0.000
Parahippocampal gyrus	27	Left	−17	−39	6	123	8.77	0.000
Thalamus	-	Left	−21	−16	0	92	8.64	0.000
Precuneus	7	Right	−2	−75	38	227	8.59	0.000
Calcarine fissure	17	Right	0	−72	−5	255	8.57	0.000
Median cingulate gyrus	32	Right	12	42	26	121	8.31	0.000
Inferior frontal gyrus (triangular part)	44	Right	60	15	11	194	8.29	0.000

**Figure 2:**
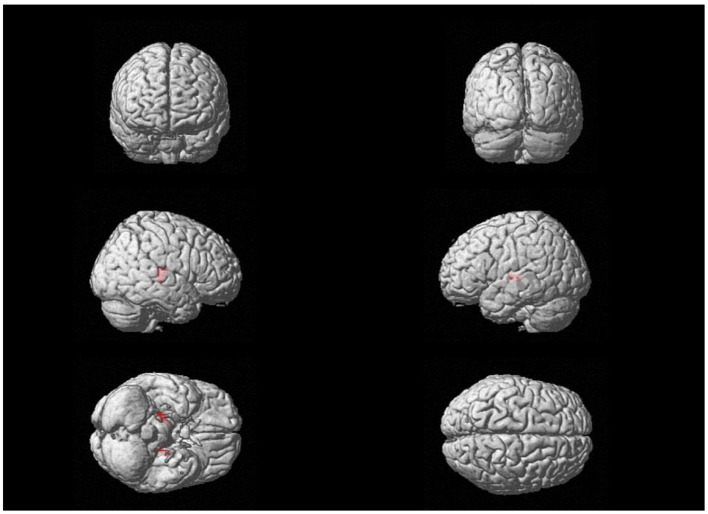
Increased gray matter volume in different brain regions in MDD patients with post-treatment vs. pre-treatment.

The abovementioned gray matter volume-reduced brain regions were used as MASK to compare the volume changes in these brain regions after treatment, and there was a significant increase in bilateral thalamic gray matter volume in MDD patients after treatment (*P* < 0.05; Table 4; Figure 2).

**Table 4:** Comparison of gray matter volume in MDD patients before and after treatment.

**Brain regions**	**Side**	**MNI coordinates**			**Cluster size**	***t***	***P***

		**X**	**Y**	**Z**			
Thalamus	Right	24	−24	8	445	6.17	0.000
Thalamus	Left	−21	−18	0	192	4.98	0.000

*F*_(2,86)_ = 4.036, *P* < 0.001.

### Correlation of gray matter volumes with depressive symptoms

In the current study, the correlation between bilateral thalamic gray matter volume and HAMD-17 score after treatment was analyzed, but the results showed no marginal significance (*p* > 0.05).

## Discussion

In the current study, the left dorsolateral prefrontal lobe was selected as the treatment target, and the depressive symptoms of MDD patients were effectively alleviated after 15 days of rTMS treatment. In order to further explore the underlying neural mechanisms of behavioral changes, this study utilized VBM analysis and found that the gray matter volumes in the right fusiform gyrus, left and right inferior frontal gyrus (triangular part), left inferior frontal gyrus (orbital part), left parahippocampal gyrus, left thalamus, right precuneus, right calcarine fissure, and right median cingulate gyrus were significantly reduced in MDD patients, while bilateral thalamus volumes were significantly increased after rTMS treatment (19).

The fusiform gyrus is located in the temporal-parietal lobe and constitutes a core systemic part of the neural network for face perception (20), and a meta-analysis showed that different brain regions are involved in the processing of different emotional facial expressions (21). The fusiform gyrus showed a decline in activation during a gradual increase in facial expression of pleasure and activation during a gradual increase in expression of sadness (22). The results of the present study showed that the gray matter volume of the right fusiform gyrus was smaller in MDD than in healthy controls, suggesting that there may be abnormalities in the network modulation of face perception in MDD patients.

The inferior frontal gyrus is the bottom-most gyrus of the frontal lobe of the brain (23) and belongs to the prefrontal cortex, which has three distinct regions, the opercular part, the triangular part, and the orbital part, based on the cytoarchitecture (24). The inferior frontal gyrus plays a decisive role in language production and processing, learning, and memory (25). It has been shown that patients with MDD often have abnormalities in the inferior frontal gyrus and that the pars orbitalis of the inferior frontal gyrus is associated with a variety of psychological functions involving social interaction and language communication (26), and the pars triangularis of the inferior frontal gyrus is associated with auditory comprehension (27). Therefore, patients with MDD have reduced gray matter volume in this area, which may be related to clinical symptoms such as reluctance to communicate with people in MDD patients.

The precuneus has a large number of neural connections with the hippocampus and the thalamus (28) and is involved in the encoding and extraction of situational memory (29). Fletcher et al. found that the precuneus is an important neural basis for processing visual images in conscious memory and is closely related to emotion regulation (30). The precuneus locates in the medial parietal lobe, which regulates the binding between 5-hydroxytryptamine and receptors. Some scholars have found that parietal injury could cause a decline in cortical 5-hydroxytryptamine receptor function, while patients exhibit a depressive state (31), indicating that a smaller volume of the precuneus gray matter is associated with the development of the depressive disorder.

Neuroimaging findings have converged on thalamic involvement in macroscopic structural abnormalities in depression (32). Morphometric abnormalities have been observed across gray matter volume (GMV) reduction in the thalamus in MDD (33). Specifically, the thalamic volume reduction has been implicated in the pathophysiology of MDD and has been associated with the severity of depressive symptoms (34). Therefore, the reduced thalamus volume is considered to be a potential marker of MDD (35). Zhang et al. manifested that the gray matter volume of the bilateral thalamus was reduced (36); however, in the current study, only the left thalamic gray matter volume was reduced. This may be related to the participants receiving drug treatment, and in Zhang's study, most participants took medication for more than 1 month (36). Some studies have found that taking medication for more than 1 month may have an impact on the brain structure (37). Therefore, the use of medication may be one of the reasons for the differences in results.

This study investigated volumetric changes in the thalamus of drug-naive depressed patients before and after 15 days of rTMS treatment. The thalamus plays a critical role in the relay and distribution of afferent signals (38), with reciprocal connections to cortical and subcortical regions facilitating subcortical-cortical information exchange (39). High-frequency rTMS has been shown to modulate cortical excitability (40) and induce long-term potentiation (LTP), a key mechanism in the therapeutic effects of TMS, which enhances synaptic strength and can persist for extended periods (41). The current study targeted the left dorsolateral prefrontal lobe with 10 Hz high-frequency stimulation, resulting in enhanced neuroplasticity in the target-associated brain regions. Given the anatomical interconnectivity between the PFC and the thalamus, a potential increase in thalamic gray matter volume following rTMS treatment could occur *via* neuroplastic mechanisms. Several studies have demonstrated that an increase in thalamic gray matter volume may ameliorate depressive symptoms (42). However, no significant correlation between thalamic volume alterations and depressive symptoms was observed in this study.

## Limitations and conclusion

The present study has some limitations that should be taken into account. First, the sample size was relatively small, which might have resulted in inadequate statistical power to detect potential group differences in behavior. A larger sample size might be necessary to clarify the group effects on rTMS treatment (43). Moreover, venlafaxine is the first of the SNRIs that provides dose-dependent norepinephrine reuptake inhibition; a dosage of 150 mg/day or higher is sufficient to produce noradrenergic activity, and it has low affinity for the postsynaptic receptors (44, 45). The latest review has demonstrated that brain function and structure changes were seen at least 4 weeks after antidepressant pharmacotherapy in patients with MDD (37). In the current study, venlafaxine, which was increased from 75 to 150 mg/day within 15 days during the rTMS treatment, may marginally affect the brain function, but a *post-hoc* test is difficult to control the confusion between the drug and the rTMS treatment results.

In conclusion, we compared changes in gray matter volume in MDD patients before and after rTMS treatment. Before treatment, MDD patients had structurally altered gray matter in the brain compared to healthy controls, which involved multiple brain regions including the frontal, temporal, and parietal lobes. These results demonstrated that brain alterations in depressive disorder are not single brain region alterations (46). No additional changes were found in gray matter volume after rTMS treatment, which may be linked to the short treatment duration. In future studies, we will expand the sample size, increase the homogeneity of patients in each group, and further explore the neural mechanisms of rTMS antidepressants in MDD patients.

## Data availability statement

The raw data supporting the conclusions of this article will be made available by the authors, without undue reservation.

## Ethics statement

The studies involving human participants were reviewed and approved by the Ethics Committee of the First Affiliated Hospital of Air Force Medical University (Grant No. KY20202055-F-1). The patients/participants provided their written informed consent to participate in this study.

## Author contributions

ZW, HW, and PZ conceived and designed the experiments. MG, DZ, XR, DL, and KY performed the experiments. BL, MG, and ZW analyzed the data. HW and PZ conceived the project and modified the manuscript. All authors have read and approved the final manuscript.
